# DNA metabarcoding quantifies the relative biomass of arthropod taxa in songbird diets: Validation with camera‐recorded diets

**DOI:** 10.1002/ece3.8881

**Published:** 2022-05-06

**Authors:** Yvonne I. Verkuil, Marion Nicolaus, Richard Ubels, Maurine W. Dietz, Jelmer M. Samplonius, Annabet Galema, Kim Kiekebos, Peter de Knijff, Christiaan Both

**Affiliations:** ^1^ 3647 Conservation Ecology Group Groningen Institute for Evolutionary Life Sciences (GELIFES) University of Groningen Groningen The Netherlands; ^2^ 3647 Groningen Institute for Evolutionary Life Sciences (GELIFES) University of Groningen Groningen The Netherlands; ^3^ Department of Human Genetics Leiden University Medical Centre Leiden The Netherlands; ^4^ Present address: Institute of Evolutionary Biology University of Edinburgh Edinburgh UK

**Keywords:** Arthropoda, COI primers, DNA barcoding, *Ficedula hypoleuca*, Illumina sequencing, insectivorous diet, PCR‐based, validation

## Abstract

Ecological research is often hampered by the inability to quantify animal diets. Diet composition can be tracked through DNA metabarcoding of fecal samples, but whether (complex) diets can be quantitatively determined with metabarcoding is still debated and needs validation using free‐living animals. This study validates that DNA metabarcoding of feces can retrieve actual ingested taxa, and most importantly, that read numbers retrieved from sequencing can also be used to quantify the relative biomass of dietary taxa. Validation was done with the hole‐nesting insectivorous Pied Flycatcher whose diet was quantified using camera footage. Size‐adjusted counts of food items delivered to nestlings were used as a proxy for provided biomass of prey orders and families, and subsequently, nestling feces were assessed through DNA metabarcoding. To explore potential effects of digestion, gizzard and lower intestine samples of freshly collected birds were subjected to DNA metabarcoding. For metabarcoding with Cytochrome Oxidase subunit I (COI), we modified published invertebrate COI primers LCO1490 and HCO1777, which reduced host reads to 0.03%, and amplified Arachnida DNA without significant changing the recovery of other arthropod taxa. DNA metabarcoding retrieved all commonly camera‐recorded taxa. Overall, and in each replicate year (*N* = 3), the relative scaled biomass of prey taxa and COI read numbers correlated at *R* = .85 (95CI:0.68–0.94) at order level and at *R* = .75 (CI:0.67–0.82) at family level. Similarity in arthropod community composition between gizzard and intestines suggested limited digestive bias. This DNA metabarcoding validation demonstrates that quantitative analyses of arthropod diet is possible. We discuss the ecological applications for insectivorous birds.

## Graphical abstract

This study is a field‐based validation of the application of DNA metabarcoding to quantitatively assess diets of wild‐living songbirds in temperate woodland ecosystems. The average relative read abundance of arthropod orders in fecal samples matched the scaled biomass contribution of these orders as observed on nestbox cameras. Hence, we demonstrated that in insectivorous songbirds fecal DNA metabarcoding can retrieve the relative biomass contribution of arthropod orders to the diet.

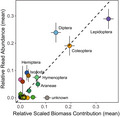

## 1 INTRODUCTION

Since its foundation, animal ecology has had a major focus on food (Elton, 1927): population abundances are often determined by food availability, and interactions among species are between predator and prey (or parasites and host), or predators competing for the same prey. However, for generalist species with complex diets, quantifying what an individual or a population consumes is a complicated task. Yet, we need quantitative methods to characterize diets to address many ecological questions, such as: (a) how do species separate their trophic niches in space and time?; (b) what are the consequences for food‐webs of global declines in major food groups, such as arthropods?; and (c) how do differential changes in prey phenology in response to global warming affect reproduction? Influential papers on the latter two topics mostly have used correlations between features of populations, species or individual phenotypes, and general indices of food availability (e.g., Both et al., 2006; Hallmann et al., 2014), often without specifying the intermediate mechanistic link with diet (but see Singer & Parmesan, 2010). When diets are examined (e.g., in songbirds: Cholewa & Wesołowski, 2011; Samplonius et al., 2016), this is often restricted to life stages when it is most easily monitored (i.e., nestlings), ignoring essential ecological and evolutionary features of predators in most life and annual stages.

DNA metabarcoding (hereafter metabarcoding) can be an important tool in ecological studies (Alberdi et al., 2019), as it allows prey detection from fecal samples and thus the establishment of longitudinal and spatial studies on trophic interactions and associated biodiversity (reviewed by Kress et al., 2015; Valentini et al., 2009; and demonstrated for an Arctic foodweb by Wirta et al., 2015). Through metabarcoding, prey taxa that are visually difficult to detect can be assessed (e.g., Ando et al., 2013), new prey taxa and feeding habitats can be discovered in relatively well‐studied species (Gerwing et al., 2016; Trevelline et al., 2018), and ecological communities can be phylogenetically described (e.g., Evans et al., 2016). Several studies have already successfully used DNA barcodes to study insectivorous diets of bats (Deagle et al., 2019; Ingala et al., 2021; Krüger et al., 2014; Zeale et al., 2011) and of birds (King et al., 2015; McClenaghan et al., 2019; Rytkönen et al., 2019; Shutt et al., 2020; Wong et al., 2015).

Relatively cheap PCR‐based sequencing protocols make metabarcoding an accessible tool for ecologists, provided that PCR primers matching a sufficient reference database are available (Taberlet et al., 2018). For arthropods, the potential of PCR‐based protocols to assess diversity is emphasized by the high detection rates of 80–90% in studies using a mix of known arthropod species (Brandon‐Mong et al., 2015; Elbrecht & Leese, 2015; Jusino et al., 2019; Krehenwinkel et al., 2018). In particular, metabarcoding of the mitochondrial Cytochrome Oxidase subunit I (COI) is being applied because this barcode is capable of retrieving every species in the assembled arthropod communities (Jusino et al., 2019; Krehenwinkel et al., 2018), and high‐quality DNA barcodes of museum reference collections are available (Hebert et al., 2003). The successful use of COI has also been demonstrated using feces of birds. Five different classes of Arthropoda and Mollusca were detected in feces of Western Bluebird *Sialia mexicana* nestlings, using generic metazoan COI primers (Folmer et al., 1994) that amplify 710 bp of the COI gene (Jedlicka et al., 2013), but this long fragment may not recover all arthropod taxa (Jusino et al., 2019). Additionally, in several warblers and Barn Swallows *Hirundo rustica*, it was shown that using a shorter COI fragment may result in more PCR product and more arthropod taxa being recovered from avian feces (Forsman et al., 2022; King et al., 2015; McClenaghan et al., 2019; Rytkönen et al., 2019). These are encouraging results for ecologists interested in metabarcoding arthropod diets from fecal samples. However, a number of issues persist.

Most importantly, it remains unknown if metabarcoding can result in a quantitative assessment of arthropod diets (Deagle et al., 2019). Many studies assess the presence/absence of taxa and not the read abundance, because PCR‐based methods may not sufficiently approximate the relative abundance of each prey taxa (Elbrecht & Leese, 2015; Piñol et al., 2015; Jusino et al., 2019, but see Deagle et al., 2019; Thomas et al., 2016). Therefore, validation tests of birds fed with recorded food items are important, especially for generalist species with more diverse diets (King et al., 2008; Pompanon et al., 2012). Also, potential biases due to differences in how taxa pass through the digestive track need to be assessed (King et al., 2008). And more technically, studies have reported PCR inhibition due to uric acids in avian feces which leads to loss of samples and jeopardizes study design (Jedlicka et al., 2013; Rytkönen et al., 2019), and difficulties in retrieving all arthropod taxa with a single PCR protocol, especially due to PCR primers mismatching with spiders (Jusino et al., 2019).

**FIGURE 1:**
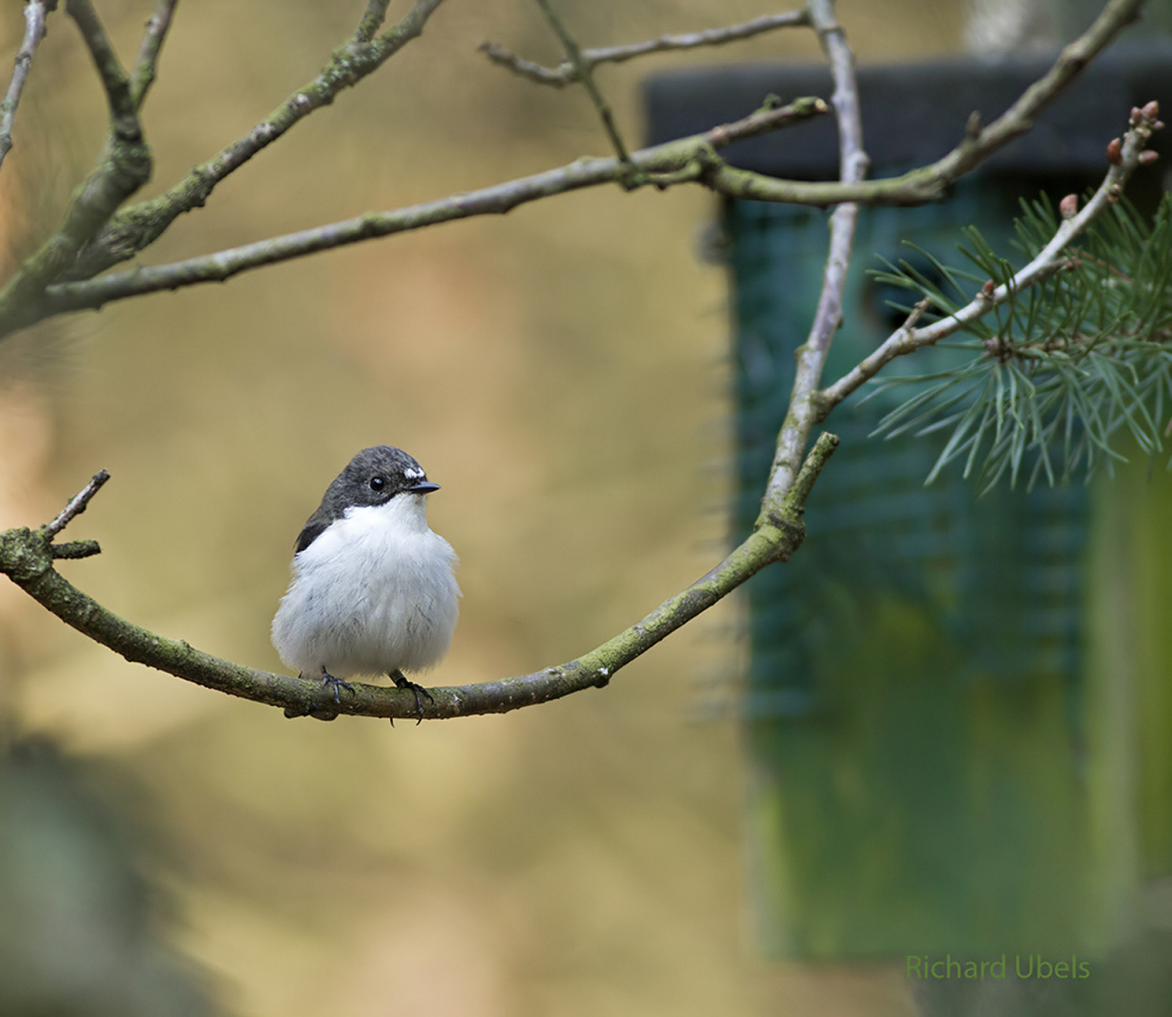
Male Pied Flycatcher *Ficedula hypoleuca* near next box in the study area in Drenthe, The Netherlands (52°49′N, 6°25′E)

This paper aims to examine whether metabarcoding can provide a quantitative estimate of the relative biomass contribution of taxa to the diet, or whether it is restricted to a qualitative assessment of the frequency of occurrence of prey in samples. We first optimize DNA‐extraction from avian feces and maximize reads of arthropod taxa with adjusted primers. We then test whether there is (a) differential loss of diet items throughout the digestive track, and (b) a quantitative match between approximated diets and the relative read number of diet taxa. Our study population of Pied Flycatchers *Ficedula hypoleuca* (Figure 1) gives the opportunity for a validation study in a natural setting. Flycatchers breed in nest boxes and provide their nestling with a large variety of taxa which is recorded on camera (Nicolaus et al., 2019; Samplonius et al., 2016) and nestling feces can be easily collected.

## 2 MATERIALS AND METHODS

### 2.1 Summary of study design

We tested the application of massive parallel sequencing, or metabarcoding, of the mitochondrial gene COI to quantify the biomass of arthropod taxa in diets in birds. First, we conducted preparatory tests to establish DNA extraction methodology, PCR primers and bioinformatics pipeline settings suitable to target Arthropoda in bird feces. Furthermore, through comparative metabarcoding of the gizzard and lower intestines content of adult Pied Flycatchers, we assessed possible effects of digestion on prey taxa detection and prey community. In the final validation test, we quantitatively compared the relative read abundance of arthropod taxa detected in feces of Pied Flycatcher nestlings with the scaled biomass prey taxa provided by their parents at the same time.

### 2.2 Field feces collection and camera observations on diet

Fecal samples were collected in the study area in Drenthe, The Netherlands from 2013 to 2017 (52°49′N, 6°25′E; see Both et al. (2016) for detailed description). For the digestion test, we used eight male adult Pied Flycatchers that were killed by Great Tits *Parus major* in a nest box between 14 April and 17 May 2015, a consequence of heterospecific competition for nest boxes (Merilä & Wiggins, 1995; Samplonius & Both, 2019; Slagsvold, 1975). For the validation test, 63 feces were collected from chicks whose food provisioning by their parents was monitored by cameras fitted inside the nest boxes (39 observation days in three years). In 2013 and 2015, fecal samples of 1–3 chicks per nest box were collected on the same day or one day before or after the camera recording day. We allowed this range of days because in this population, diet composition is repeatable between subsequent days (Nicolaus et al., 2019). However, to test whether more targeted timing is important for validation, in 2016, feces of two chicks per nest box were always collected on the same day at the end of the recording period. Samples were placed in a sterile 2.0 ml tube with 96% ETOH. Fecal sacs around feces of chicks were opened by shaking to ensure mixing of fecal material and ETOH. Samples were stored at −20°C. For long‐term storage, all samples were stored in −80°C freezers.

From camera sessions of ca. 2 h (five in 2013, 18 in 2015, and 16 in 2016), camera footage was scored, noting the food item provided and its relative size in relationship to the beak of the adult (details in Samplonius et al., 2016). To allow comparison with the read counts obtained from metabarcoding of feces which reflect ingested biomass and not prey numbers, the prey counts from camera footage were size‐adjusted to obtain the scaled biomass contribution of each taxa. With this adjustment, large prey items were given extra weight compared with small prey, by using a multiplication factor varying from 0.04 to 6.0 depending on the prey size relative to the bill size. Prey counts were taxonomically assigned to arthropod class, order, and if possible also to family, genus, and species, yielding taxonomically unique groups (for comparison with COI data called “camera‐OTU”) that could vary in precision of taxonomic assignment. The camera‐OTU “unknown” accumulated all counts of unclassified animals. Prey items were taxonomically assigned without prior knowledge of metabarcoding results, and no prey items were reassigned a posteriori.

### 2.3 Tests of DNA extraction methods and modified PCR primers

In a double pair‐wise design, fecal samples of two nestlings were divided over two DNA‐extraction methods, and for each extraction method, two different PCR primer pairs were tested, creating four replicates of pair‐wise comparisons of methodology. The tested DNA extraction methods were (a) Qiagen DNeasy PowerSoil Kit, formerly made by MoBio, or (b) Invitrogen™ PureLink™ Microbiome DNA Purification Kit. Following the successful application in insectivorous songbirds (King et al., 2015), we tested the generic invertebrate COI primers LCO1490 (Folmer et al., 1994) and HCO1777 (Brown et al., 2012) and designed modified versions LCO1490_5T and HCO1777_15T (Table 1). These modified primers contain a mismatch with the host but not with the main Arthropod orders, as assessed including the following taxa/sequences (GenBank accession in brackets): Ficedula, Passeriformes (GU571891), Araneae (KP655120), Coleoptera (KF317270, KM447512), Diptera (KF297873, KP037602), Hemiptera (KM021773, KJ541653, KM022933) Hymenoptera (KX665113, AB007981), and Lepidoptera (GU654879, JF860168, FJ412377).

**TABLE 1:** The generic invertebrate COI primers, and the modified version. In the primer sequences, the new variations are indicated in bold

COI primer	Forward	Sequence	Source
Original	LCO1490	5′‐GGTCAACAAATCATAAAGATATTGG‐3′	Folmer et al. (1994)
Modified	LCO1490_5T	5′GGTC**T**ACAAATCATAAAGATATTGG‐3′	This study
	Reverse		
Original	HCO1777	5′‐ACTTATATTGTTTATACGAGGGAA‐3′	Brown et al. (2012)
Modified	HCO1777_15T	5′‐ACTTATATT**A**TTTATACGAGGGAA‐3′	This study

Note

Note that nomenclature refers to the base on the locus.

### 2.4 Laboratory procedures

Fecal samples were subsampled to arrive at a sample weight of <1 g to reduce levels of uric acids, which are present in bird feces and cause PCR inhibition (Jedlicka et al., 2013). DNA was extracted with the Invitrogen™ PureLink™ Microbiome DNA Purification Kit, after establishing in the replicated pair‐wise test that this method yielded consistently more reads than the Qiagen DNeasy PowerSoil Kit (see Section 3). For both methods, the manufacturer's protocol was altered as follow: (a) a bead beater was used instead of a vortex mixer, in 5 × 2 min bouts pausing 30 s between bouts, (b) approx. 0.1 g extra 0.1 mm Zircona/Silica beads were added, (c) for the final elution, 20 µl Ambion© purified DNA‐free water was used, and (d) pre‐elution incubation was extended to 4–5 min and DNA was re‐applied to the filter and incubated 2 min extra before final elution.

To prevent contamination, all materials were autoclaved and UV‐sterilized for 20 min before use. Two negative control extractions with no fecal sample were included to test the purity of the extraction kits and to test for cross‐contamination between samples during the extraction procedure. DNA concentrations were not normalized before PCR because fecal samples contain more bacterial and host DNA than target prey DNA.

PCRs were set up in a DNA‐free room. Each sample was amplified in duplicate (pair‐wise test) or triplicate (digestion and validation test) to avoid PCR bias, and from each PCR master mix, negative controls were taken to track possible contamination of PCR reagents. Before pooling, negative controls were assessed with 5 µl PCR product in a standard gel electrophoreses. Annealing temperature was set low to minimize taxonomic bias (following Ishii & Fukui, 2001).

PCRs had a final reaction volume of 20 µl containing 2.5 µl 10x Roche buffer, 0.2 µl 25 mM dNTPs, 0.88 µl 50 mM MgCl2, 0.03 µl BSA, 1.0 µl of each primer (10 μM), 0.2 µl 5 U/μl Taq polymerase (Roche), and 5 μl DNA template. The PCR profile included an initial denaturation at 94°C for 2.5 min., 35 cycles of 94°C for 30 s, 48°C for 30 s and 72°C for 45 s, and a final extension at 72°C for 10 min. For eight of 16 gizzard/intestine samples, AccuStart II PCR ToughMix© was used to improve the amplification success, as DNA may have been degraded, because samples were taken from birds that may have been dead for a day before they were found in the nest box. The reaction volume was 10 μl including 5 μl AccuStart, 1μl of each primer (10 μM), 1 μl ddH2O, and 2μl DNA template. When using AccuStart, the PCR profile was altered to 3 min. at 94°C followed by 35 cycles of 1 min at 94°C, 30 s at 48°C and 1 min at 72°C, and a final extension at 72°C for 10 min.

### 2.5 Massive parallel sequencing

PCR products (total *N* = 16 + 17 + 63 = 96) and the pooled negative extraction controls (*N* = 2) were sequenced on the MiSeq© Sequencer (Illumina) at the Department of Human Genetics, Leiden University Medical Centre, aiming for a read depth of 50,000 per sample. Libraries were prepared with the MiSeq© V3 kit, generating 300‐bp paired‐end reads. The V3‐kit does not normalize, that is, leaves the relative presence of initial PCR product intact, and therefore, this library preparation method allows assessing the relative contribution of prey taxa. Eight PCR products were sequenced twice: in a limited run aiming for 10,000 raw reads per sample and an extended run aiming for >50,000 raw reads per sample. With a rarefaction plot, we assessed the detected number of total taxa and arthropod taxa against sequencing depth.

### 2.6 Bioinformatics pipeline design

Using the software USearch 9.2 (Edgar, 2010), we extracted unique high‐quality barcode reads (molecular operational taxonomic units, abbreviated as OTU). First, paired raw reads (the forward and reverse reads) were merged, to obtain a consensus sequence. This removed unaligned segments at both ends which contained sequencing adaptors. Primer sequences were removed by truncating each end by 25 bp, the length of the longest PCR primer.

Effects of the next steps, quality filtering and read truncating, were first empirically tested (Alberdi et al., 2018). We tested 18 combinations of settings on the pooled data of five fecal samples (sequencing ID: T0113, T0218, T0313, T0420, and T0520). The error (E) values varied between 0.1 and 1.0 (while truncating at 220 bp) where *E* = 1 means all reads, including low quality reads, are included, and decreasing E‐values means more stringent filtering. Truncation values varied between 140 and 280 bp (while filtering at 0.4). Differences between settings were statistically tested by a One‐Way Repeated Measures ANOVA on number of reads (abundance) per arthropod taxa, using the single Anova(mod, idata, idesign) function in *R* package *car* (Fox et al., 2013). The dependent variable *mod* was the linear model correlating reads per taxa between the 18 variants, *idate* was our dataframe taxa abundance, and the factor *idesign* was our pipeline variant. In each pipeline on average 501,580 (493,516–505,543), paired reads were obtained. Arthropoda had on average 493,203 (486,100–497,380) reads. Between pipeline settings, taxonomic assignment to class, order, and family did not vary significantly (*F*_1,17_ = 0.17, *p* = .99). The diversity indices showed a slight optimum when trimming was set to 180–220.

For the full dataset, filtering was set at default E‐value of 0.4 and read truncation to 220 bp. To simplify clustering, truncated reads were de‐replicated assigning a count to unique reads. This also merged identical reads that are present in both orientations. Subsequently, the singletons were removed. Using the UPARSE‐OTU algorithm (Edgar, 2010), reads that were minimally 97% identical were clustered, and the consensus sequence of each cluster was assigned an OTU ID; this created an OTU sequence database. This algorithm also filters chimeras.

Lastly, for each sample, the number of reads (paired and with primers truncated) that matched with each OTU was determined, resulting in an OTU frequency table. The default identity match of 97% was used. This setting was evaluated by comparing OTU tables created with 90% and 97% identity matches in the validation test; as values highly correlated, we used the more stringent matching with a 97% cut‐off which was possible without data loss.

The final OTU frequency table was adjusted for the pooled negative extraction and PCR controls, by deducting the number of reads found for an OTU in the pooled negative extraction controls from each cell in the OTU table. The sum of reads in the pooled negative controls was 70 reads with a maximum of 10 per OTU (Table S1).

### 2.7 Taxonomic assignments

The obtained OTU databases were searched against the *nt* database in GenBank (Benson et al., 2009), using the BLAST function in Geneious 8.1.7 (Kearse et al., 2012). We used GenBank, which also contains the public part of sequences from BOLD (Barcode of Life Data Systems) (Ratnasingham & Hebert, 2007), because species diversity of the Western European arthropods is sufficiently covered (King et al., 2008). Also, validating our approach with a public database will demonstrate the general applicability for other European studies.

We used the Megablast option which is faster than blast‐n and only finds matches with high similarity. Settings were as follows: max *e*‐value = 1*e*‐1 (the lower the number of expected (*e*) hits of similar quality the more likely the hit is real), word size = 28 bp (minimal match region), and gap cost = linear. The best hits were saved in a query‐centric alignment. For each match between an OTU and a reference organism, we recorded the following: non‐annotated matching sequences, query coverage, bit‐score, *e*‐value, pair‐wise identity, sequence length and grade. The grade is a percentage calculated combining three statistics: the query coverage, *e*‐value, and pair‐wise identity values for each hit, which have a weight in the equation of 0.5, 0.25, and 0.25, respectively. Each encountered reference organism was included in a taxonomy database with its GenBank Accession number. Inclusion of a reference organism in our taxonomy database was independent of the likelihood of occurrence in the study area (i.e., in some cases an OTU matched best with a species not occurring in Europe).

Taxonomic categories included were Kingdom, Phylum, Class, Order, Family, Genus, and Species. The utility of assignment to the order and family level was explored in the validation test. Digestive biases were assessed at the genus level. The grade score was used to assess the reliability of taxonomic assignment. We considered species assignments only indicative of the actual species. In general, species assignments through a similarity match of short OTU reads to reference sequences is ambiguous: even when an OTU has a 100% match with a species barcode, the probability that it is the same species is not 100% (Ward, 2009).

### 2.8 Data analyses

Data analyses were performed in *R*, using packages *phyloseq* (McMurdie & Holmes, 2013) and *vegan* (Dixon, 2003). The data were pruned to the target phylum Arthropoda, and read counts of taxa in each sample were transformed to relative read abundance (RRA) on the order or family level. To arrive at a higher aggregate scale (e.g., year), average RRA (±*SD*) per taxa per sample was calculated (step 1: percentage per sample, step 2: average across these percentages). Prevalence was expressed as the number of samples in which taxa occurred (Frequency of occurrence ‐ FOO). In the camera records, to create diversity statistics, the scaled biomass had to be rounded to integers.

The gizzard‐intestine differences in the FOO of genera were tested with a contingency Chi square analysis; this analysis was restricted to common taxa, occurring in ≥3 samples. NMDS ordination was performed in *phyloseq* on OTU level (because at an average grade of 99% taxonomic assignments were sufficiently reliable) with categorical variables sample type and bird ID; read counts per sample were normalized to median count, and singletons were pruned to reach convergence. To assess whether community variability is better explained by sample type (gizzard or intestine) or individual, a *permutest* was conducted.

Arthropod community differences between fecal samples collected during different camera sessions were assessed with ordination analyses, applying nonmetric multidimensional scaling (NMDS), and using the Bray–Curtis distance to calculate beta dispersion, and analyses of variance on the distance matrix (*Adonis*; which uses pseudo‐*F* ratios) (Anderson, 2001). We tested the null hypothesis of no difference in dispersion between camera sessions, and report on sum of square of sessions and residuals to explore how much of the community variability is explained by camera session as opposed to replicate samples within session.

Pearson's correlation tests were applied to evaluate which metric of COI read counts better described the camera‐recorded scaled biomass of taxa, FOO or RRA. In contrast to rank correlations, Pearson's correlation test allow to explore differences in the linearity of relationships quantitatively. The variation in the correlations between single feces/camera‐session combinations was not explored with multilevel models because (a) to include FOO in the comparison aggregated data had to be used, (b) no predictor values exists because of uncertainties in scaled biomass estimates from camera footage. For these analyses, the parasites orders Mesostigmata, Prostigmata, Sarcoptiformes, Siphonaptera, and Trombidiformes were excluded because they likely were inside other prey as parasitic larvae.

## 3 RESULTS

### 3.1 DNA extraction, PCR primers, and sequencing depth

We introduced miss‐priming with flycatcher DNA while improving the yield of spider DNA, and we established a target sequencing depth of 2000–10,000 paired reads per sample.

The four replicate sample pairs yielded 219,241 paired reads assigned to 424 OTUs of which 192,228 reads were Arthropoda, assigned to 244 OTUs, divided over five classes, 17 orders and 64 families and 101 genera. In each pair, DNA‐extraction with PureLink yielded more reads than extraction with PowerSoil (5121–12,443 extra across replicas), and also more reads assigned to Arthropoda (2,547–11,828 extra across replicas). Within each pair, the RRA of arthropod orders correlated between the original and modified primers at *R* ^2^ = .98, *R* ^2^ = .98, *R* ^2^ = .72 and *R* ^2^ = .99, respectively (Table 2). The sum of reads assigned to Chordata (mostly Aves) was 12,933 reads (6%) with the original primers, and 3 reads (0.001%) with the modified primers. Overall, no differences were observed between primer pairs in the RRA of arthropod orders (*R* ^2^ = .92, *p* < .001), but the modified primers yielded more Araneae reads (5.6%) than the original primers (1.7%). Also, in Hemiptera (true bugs) and Hymenoptera (mostly ants and some parasitoid wasps), there were small differences in the same direction between all four replicates (Table 2).

**TABLE 2:** Pair‐wise comparison of RRA per arthropod order obtained with original or modified primers, using DNA template of either of the two DNA extraction methods

Orders	Original	Modified	Original	Modified	Original	Modified	Original	Modified
Araneae	3.01%	8.46%	**1.82%**	**7.98%**	2.09%	5.99%	**0.03%**	**0.14%**
Coleoptera	0.92%	0.08%	**0.81%**	**0.58%**	46.11%	23.28%	**1.73%**	**0.36%**
Collembola	0.51%	2.10%	**0.55%**	**1.71%**	0.00%	0.00%	**0.00%**	**0.00%**
Diplostraca	0.00%	0.00%	**0.00%**	**0.00%**	0.00%	0.00%	**1.11%**	**0.00%**
Diptera	2.08%	3.71%	**7.56%**	**5.14%**	2.95%	8.09%	**0.84%**	**0.20%**
Hemiptera	53.74%	47.24%	**64.20%**	**58.17%**	2.38%	1.60%	**1.11%**	**0.11%**
Hymenoptera	11.47%	13.33%	**8.91%**	**10.43%**	37.43%	55.46%	**90.35%**	**98.96%**
Isopoda	4.98%	3.88%	**5.85%**	**3.37%**	0.00%	0.00%	**0.00%**	**0.00%**
Lepidoptera	21.81%	19.41%	**9.53%**	**12.40%**	0.02%	0.11%	**0.40%**	**0.08%**
Mesostigmata	1.43%	1.40%	**0.55%**	**0.04%**	0.60%	0.00%	**4.31%**	**0.01%**
Neuroptera	0.00%	0.00%	**0.00%**	**0.00%**	0.00%	0.00%	**0.00%**	**0.00%**
Orthoptera	0.00%	0.00%	**0.00%**	**0.00%**	0.00%	0.05%	**0.00%**	**0.00%**
Prostigmata	0.01%	0.00%	**0.00%**	**0.00%**	6.57%	4.93%	**0.06%**	**0.13%**
Psocoptera	0.01%	0.40%	**0.09%**	**0.14%**	0.00%	0.00%	**0.00%**	**0.00%**
Thysanoptera	0.03%	0.00%	**0.12%**	**0.05%**	0.00%	0.00%	**0.00%**	**0.00%**
Trichoptera	0.00%	0.00%	**0.00%**	**0.00%**	0.00%	0.00%	**0.00%**	**0.00%**
Trombidiformes	0.00%	0.00%	**0.00%**	**0.00%**	1.86%	0.49%	**0.06%**	**0.01%**
	S1 PowerSoil		**S1 PureLink**		S2 PowerSoil		**S2 Purelink**	

Note

Given are RRA per order. S1 and S2 refer to sample IDs. Extraction methods were PowerSoil (Qiagen DNeasy *PowerSoil* Kit) and PureLink (Invitrogen™ *PureLink*™ Microbiome DNA Purification Kit; in bold). Each DNA extraction was tested with the original and modified primers. Original primers: LCO1490‐HCO1777; modified primers: LCO1490_5T‐HCO1777_15T (see Table 1).

**FIGURE 2:**
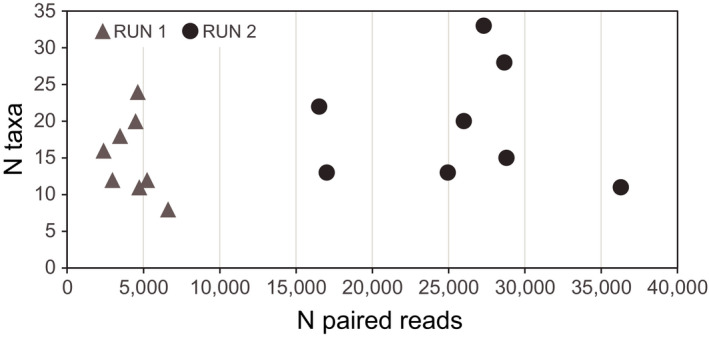
Rarefaction plot for number of arthropod taxa found against sequencing depth in the limited sequencing run: RUN 1, approximately 10,000 raw sequences per sample and 2400–6700 paired reads, and the extended sequencing run: RUN 2, >50,000 sequences per sample yielding 16,500–36,300 paired reads. Parasites were excluded to assess the effect of read depth on food taxa

The median number of arthropod reads per PCR product was 25,112 (range 15,133–36,564; merged runs). The sequencing depth of the replicas that were sequenced in the limited (*n* = 8) versus extended Illumina run (*n* = 8) was 9777–11,908 versus 50,056–80,113 raw reads (2400–6,700 and 16,500–36,300 paired reads). In this range, no effect of read depth was found on the number of arthropod OTUs (Figure 2).

### 3.2 Digestive bias

We established no directional difference in prey community and taxa abundance between gizzard and intestines of individual adult flycatchers.

For the eight paired gizzard and intestines samples (*n* = 17 including a PCR replicate), we obtained 887,188 reads and after subtraction of reads found in negative controls, 886,749 reads (12,171 to 92,334 reads per sample) which were assigned to 258 OTUs. The target phylum Arthropoda had 797,422 reads; the second largest group were parasitic worms found in one intestine sample (phylum Acanthocephala; 60,281 of 86,927 reads). The median number of reads per sample assigned to arthropods was 46,127 (range 11,431–91,418), divided over 12 orders, 53 families, and 81 genera. OTU richness and Shannon‐diversity did not correlate with the number of reads (resp. *R* = .25 (CI −0.26 to 0.65); *p* = .33 and *R* = −0.32 (CI −0.69 to 0.19); *p* = .21). All genera had high taxonomic assignment grades of on average 99% (90–100%) with an outlier of 49% for one genus (Diploplectron).

**FIGURE 3:**
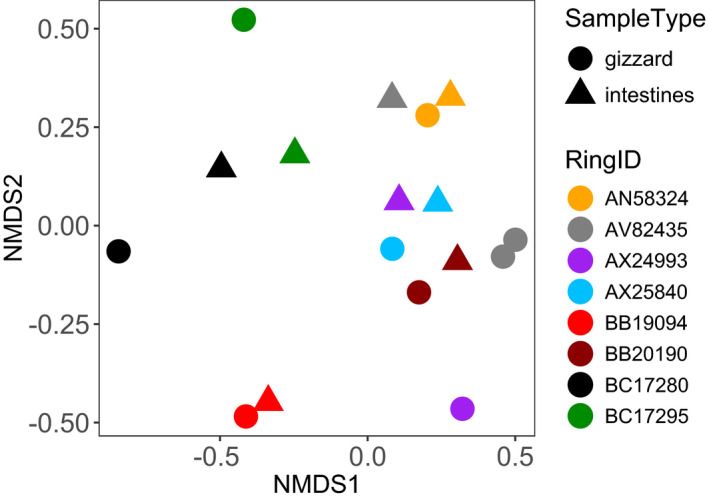
Arthropod taxa community (OTU level) in gizzard and intestinal samples of adult Pied Flycatchers represented by a Bray‐Curtis ordination plot of paired samples types, color‐coded by each bird's ring ID (metal band number); note that for bird AV82435 (in gray) a gizzard PCR replicate is shown. After 20 runs the number of NMDS (Nonmetric Multidimensional Scaling) dimensions were reduced to 2 and stress was 0.183

Overall, arthropod communities were more similar within an individual than between individuals although patterns varied between birds (Figure 3; permutest (gizzard vs. intestine): *F* = 0.053, *p* = .82; permutest (Bird ID): *F* = 1.039: pair‐wise *p* ≤ .01 (21 pairs), *p* > .1 (seven pairs)). Also, the FOO of common taxa was not significantly different between organs (*X* ^2^ = 3.721, *df* = 16, *p* = 1.00). In both gizzards and intestines, FOO was highest for *Kleidocerys* (Hemiptera), *Boletina* (Diptera), *Strophosoma* (Coleoptera), and *Formica* (Hymenoptera) (Table 3). PCR replicates of the gizzard of flycatcher AV82435 had very similar arthropod communities (Figure 3).

**TABLE 3:** Arthropod taxa community composition in gizzard and intestinal samples

Order	Family	Genus	Putative species	Grade	FOO Gizzard	FOO Intestines
Hemiptera	Lygaeidae	*Kleidocerys*	*Kleidocerys resedae*	0.982	8	8
Hymenoptera	Formicidae	*Formica*	*Formica sanguinea*	1	6	7
Diptera	Mycetophilidae	*Boletina*	*Boletina griphoides*	1	8	7
Coleoptera	Curculionidae	*Strophosoma*	*Strophosoma capitatum*	1	7	6
Coleoptera	Chrysomelidae	*Lochmaea*	*Lochmaea capreae*	0.902	4	5
Diptera	Chironomidae	*Procladius*	*Procladius nigriventris*	0.998	4	5
Diptera	Calliphoridae	*Pollenia*	*Pollenia amentaria*	1	6	5
Diptera	Culicidae	*Aedes*	*Aedes* sp.	1	8	5
Diptera	Limoniidae	*Limonia*	*Limonia nubeculosa*	0.975	2	4
Coleoptera	Carabidae	*Pterostichus*	*Pterostichus oblongopunctatus*	1	4	4
Neuroptera	Hemerobiidae	*Hemerobius*	*Hemerobius micans*	1	1	3
Hemiptera	Miridae	*Harpocera*	*Harpocera thoracica*	0.998	2	3
Trichoptera	Limnephilidae	*Limnephilus*	*Limnephilus auricula*	1	2	3
Diptera	Drosophilidae	*Phortica*	*Phortica* sp.	0.973	3	3
Diptera	Tachinidae	*Campylocheta*	*Campylocheta praecox*	1	4	3
Diptera	Chironomidae	*Chironomus*	*Chironomus* sp.	1	4	3
Hymenoptera	Diprionidae	*Gilpinia*	*Gilpinia virens*	0.998	4	3

Note

Given are putative species and their taxonomy, grade (quality of match with the GenBank reference sequence) and FOO (frequency of occurrence) in either sample type. Only genera occurring FOO ≥3 in at least one sample type are shown.

### 3.3 Camera‐recorded nestling diet

**FIGURE 4:**
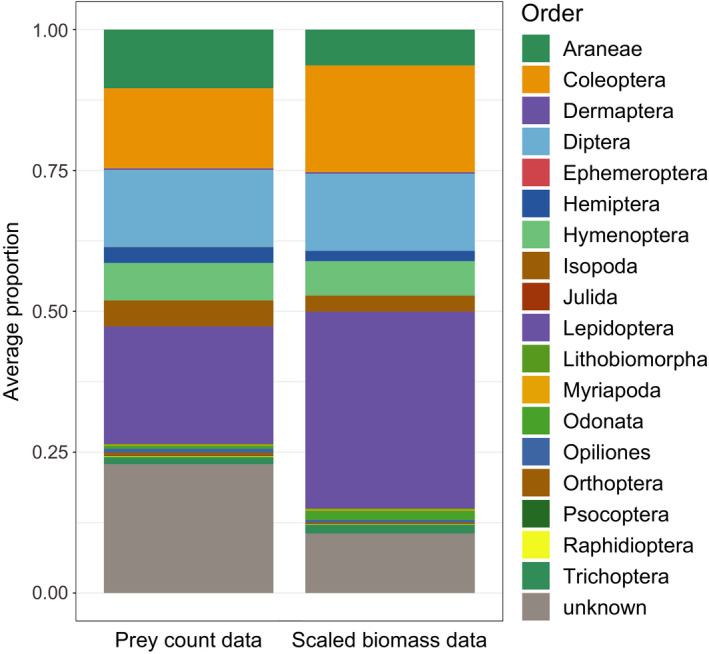
Counts of prey items versus the scaled biomass contribution of prey items in the camera records. The biomass contribution of each prey item was estimated by scaling the size relative to bill size. The stacked bars show the average relative abundance of each detected order based, averaged over 39 camera sessions

Prey items in the camera records were divided over 124 taxonomically unique groups belonging to four arthropod classes: Arachnida, Insecta, Diplopoda, and Malacostraca. In the 39 camera sessions, a total of 7314 food items were counted. The median number of food items observed per camera session was 118 (range 61–468). A total of 123 taxonomically unique groups (for comparison with COI data called camera‐OTU) were identified on order level (18 orders), 105 on family level (59 families), and 46 on genus level (40 genera). One camera‐OTU contained the accumulated 1,040 counts (9.9%) of “unclassified animals.” Camera‐OTU richness in camera sessions varied significantly with the number of observed food items (*R* = .36 (CI 0.05–0.61); *p* = .02) but Shannon‐diversity did not (*R* = .31 (CI −0.01 to 0.57); *p* = .06). Scaling prey items in size relative to bill size—to obtain the scaled biomass contribution of each food item—especially increased the relative importance of Lepidoptera (Figure 4).

### 3.4 Metabarcoding of nestling diet

The COI barcode dataset was reduced to the five arthropod classes—Arachnida, Insecta, Chilopoda, Diplopoda, and Malacostraca to match the classes found in the camera records. Chilopoda was added because in the camera footage analyses the camera they may have been lumped with the morphologically very similar Diplopoda. In the 63 fecal samples of nestlings, these five classes represented 98.4% of the data amounting to 897,315 of 912,130 assigned reads, which after subtraction of negative control reads was reduced to 911,947 reads. The dataset contained 832 OTUs, covering 24 Arthropod orders.

In the fecal samples, overall 145 arthropod families were detected and 297 genera which had a median assignment grade of 99.9 (mean 98.2). For nine genera, the grade score was below 90; they were represented by 232 reads (2–119 reads per sample). A total of 43 genera had grades of 90–97 suggesting that the actual genus may not have been available on GenBank. The three most abundant OTUs, present with >50,000 reads (max. 93,244), were assigned to *Panolis flammea* (Lepidoptera, Pine beauty), *Kleidocerys resedae* (Hemiptera, birch catkin bug), and *Porcellio scabe*r (Isopoda, common rough woodlouse).

**FIGURE 5:**
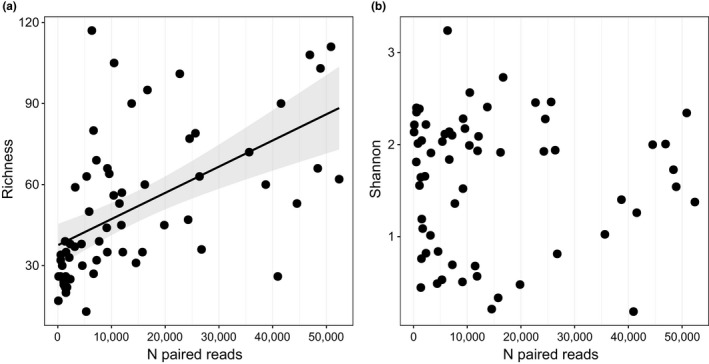
Diversity indices of the COI metabarcodes versus sequencing depth ‐ total number of paired Arthropod reads per sample. (a) Richness: number of arthropod OTUs detected per sample. (b) Shannon‐diversity. Here, the reduced data set was used (897,315 reads) including five arthropod classes (Arachnida, Insecta, Chilopoda, Diplopoda and Malacostraca) which covered 98.4% of all data

The median number of reads per sample was 9,119 (range 108–52,573); in six samples <1000 reads were assigned. The read depth per sample correlated with the observed number of arthropod OTUs (*R* = .56 (CI 0.35–0.71); *p* < .001), but not with Shannon‐diversity (*R* = −.03 (CI −0.28 to 0.22); *p* = .83; Figure 5). In eight of 63 fecal samples >90%, reads were of a single order, mostly represented by a single genus; samples were dominated by Diptera (*n* = 3), Hymenoptera (*n* = 2), Hemiptera (*n* = 1), Coleoptera (*n* = 1), or Lepidoptera (*n* = 1).

### 3.5 Validation test: retrieving the relative biomass contribution of taxa

We validated that the RRA of taxa in fecal samples can approximate the relative biomass of consumed taxa. To compare COI barcodes with camera records from the same broods, 4 of the 63 samples were discarded for low read quality or missing camera records, leaving a dataset of 59 fecal samples and 39 camera sessions. Obtained sample sizes for camera sessions versus COI barcoded feces were as follows: 2013: 5 vs. 5, 2015: 18 vs. 24, 2016: 16 vs. 30. In camera sessions with duplicate samples (*n* = 19), variation in communities detected with COI barcodes was mostly explained by camera session (Order: *F* = 1.68, *p* = .012, Mean Sq(Camera ID) = 0.33, Mean Sq(residuals) = 0.20; Family: *F* = 1.33, *p* = .003, Mean Sq(Camera ID) = 0.47, Mean Sq(residuals) = 0.35).

**FIGURE 6:**
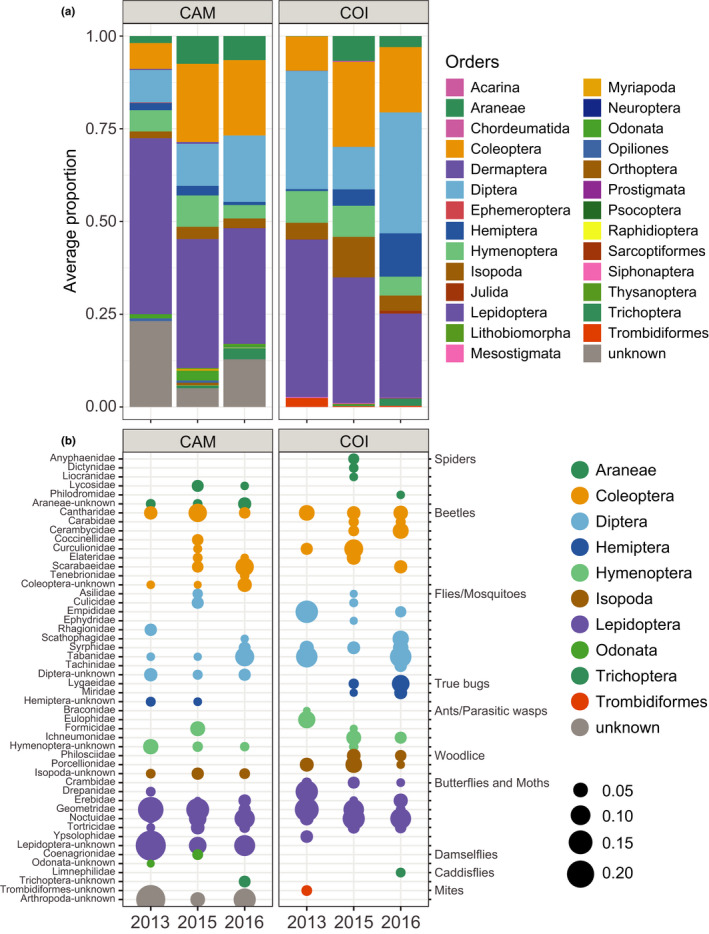
Average proportion of arthropod taxa in camera sessions and in fecal DNA. CAM = camera sessions with the proportion of scaled biomass. COI = COI metabarcodes in feces collected during camera sessions with the proportion of reads. Visualized is the average proportion of a taxa for (a) orders and (b) families. Panel b shows a reduced dataset of families present with an average proportion >0.01 and the category “unknown,” color‐coded by order

In the validation dataset, COI barcodes contained 22 orders versus 18 in the camera records; the same orders were abundant in both datasets (Figure 6a). Within the six most abundant orders, two times more families (105 vs. 50) were detected in COI barcodes than on camera images: Diptera (33 families: 31 vs. 13), Lepidoptera (22: 18/9), Coleoptera (16: 15/10), Hymenoptera (15: 13/6), Hemiptera (11: 10/4), and Araneae (18: 18/8). On family level, per camera session on average 39% of the items were not assigned to family, and hence unknown (Figure 6b). On the sample level, the number of arthropod taxa detected in the COI barcodes was significantly higher than in camera records, both at the order level (*F* = 72.53, *p* < .001) and family level (*F* = 84.33, *p* < .001).

**FIGURE 7:**
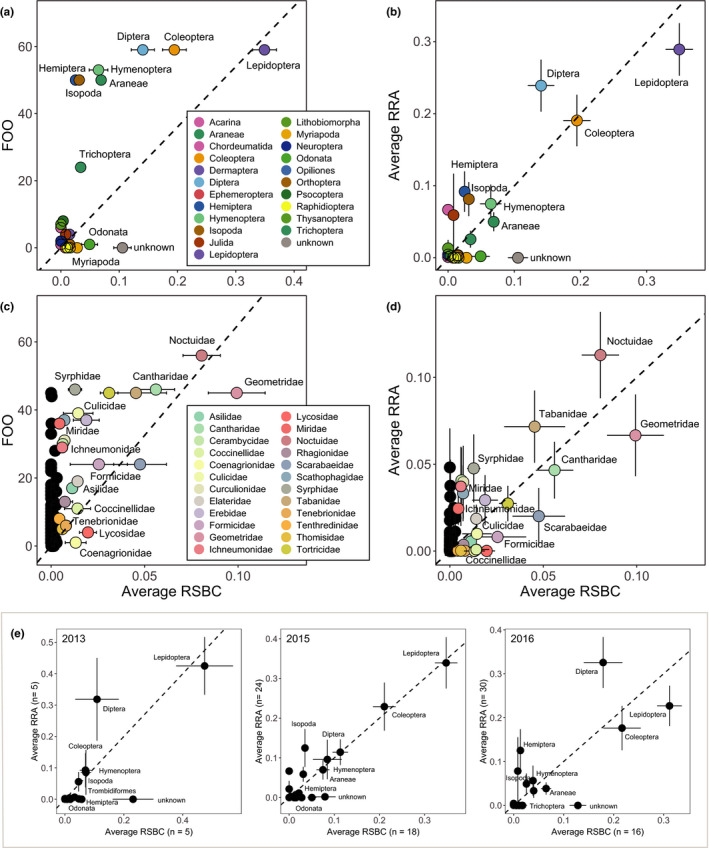
Validation test of retrieval of scaled biomass contribution of arthropod taxa through metabarcoding of pied flycatcher feces. Biomass contribution estimates of taxa in the diet are based on nest box camera footage. For the aggregated study years, the top row depicts results for arthropod orders (a, b), and the middle row for arthropod families (c, d; in black families rarely or never recorded on camera). The bottom row (e) shows each study year separately on the orders level. FOO = frequency of occurrence over all samples; RRA = relative COI read abundance of taxa in a fecal sample; RSBC = relative scaled biomass contribution of each taxa in a camera session. Plotted are FOO (a, c) and average RRA (b, d) for 59 fecal samples versus average RA in 39 camera sessions. The dashed lines illustrate the X = Y relationship to guide the eye. Common taxa are labeled

On the order level, the average RRA of taxa in fecal samples correlated strongly with the scaled biomass detected on camera (*R* = .85 (95CI: 0.68–0.94), *p* < .0001). The FOO of taxa showed a weaker correlation (*R* = 0. 65 (95CI: 0.33–0.84), *p* = .0007), which appears nonlinear as a result of overestimating Araneae, Isopoda, Hemiptera, and Hymenoptera (Figure 7a,b). Also on the family level, the correlation with scaled biomass on camera was stronger for RRA (*R* = .74 (0.66–0.81), *p* < .0001) than for FOO (*R* = .61 (95CI: 0.49–0.71), *p* < .0001) (Figure 7c,d). Correlations of RRA with scaled biomass were consistent across three study years (Figure 7e): 2013 (*R* = .76 (95CI: 0.39–0.92), *p* = .001), 2015 (*R* = .92 (95CI: 0.80–0.97), *p* < .0001) and 2016 (*R* = .78 (95CI: 0.51–0.91), *p* < .0001).

## 4 DISCUSSION

### 4.1 Proof of principle: what we have learned?

We validated that read abundance of DNA metabarcodes in feces quantitatively approximates the scaled biomass of arthropods in the diet of an insectivorous bird at the order and family level: in all three study years, across 5–18 camera sessions per year, the relative abundance of COI reads in feces matched the scaled biomass of orders and families in the observed diet. We also established that digestion did not bias taxa recovery.

The successful validation of our metabarcoding approach opens the opportunity to quantitatively monitor arthropod diets and trophic interactions. Essential technical elements of our approach are (a) host‐avoiding, non‐degenerative primers targeting all arthropod taxa including spiders, (b) extraction methods neutralizing uric acids, (c) low annealing temperatures and triplicate PCRs for high taxonomic resolution, and (d) sequencing library preparation without normalization and cost‐effective sequencing depths of 2000–10,000 reads per sample. Before application in other study systems, we recommend local validation with recorded diets. The taxonomic level of validation can be improved by involving specific taxa experts when analyzing camera footage, or by feeding experiments or taxon‐specific PCR to establish correction factors for certain prey groups (Thomas et al., 2016; Zeale et al., 2011).

### 4.2 How important is validation?

Validation studies using mock communities or captive animals fed a known diet have been conducted for a wide range of consumers and prey. These studies have shown that broad correlations are likely, but nevertheless, biases may occur that need to be accounted for (reviewed in Deagle et al. (2019), see also Thuo et al. (2019)). Therefore, Deagle et al. (2019) recommended to incorporate cross‐validation in a study set‐up whenever possible.

We found that the approximated biomass of arthropod taxa in the diet could be quantitatively retrieved with deviations within an order of magnitude. This is in contrast with earlier studies using composed arthropod communities (Elbrecht & Leese, 2015; Piñol et al., 2015) which showed that the recovered read abundance per taxon could vary by two to four orders of magnitude from the biomass in the mock community (see also Jusino et al., 2019; Krehenwinkel et al., 2017). In Piñol et al. (2015), especially the read abundance of spiders was underrepresented. Piñol et al. (2015) used the ZJB primers developed by Zeale et al. (2011) for the COI locus in arthropods, and pointed out the high number of mismatches between spider template and primers. Underestimation of spiders with the ZJB primers has been reported before (Aldasoro et al., 2019; da Silva et al., 2019). Following King et al. (2015), we used the general invertebrate “Folmer‐Brown” primers (Brown et al., 2012; Folmer et al., 1994), which retrieved spiders much better (especially the modified primers, see Table 1). Elbrecht and Leese (2015) used the original Folmer primer pair and also attributed the discrepancy to mismatches between DNA template and primers. They reported low taxa recovery within Diptera which we did not see in our study with the modified Folmer‐Brown primers. Krehenwinkel et al. (2017) used modified ZJB and Folmer primers in various combinations but were unable to amplify some Acari and Hymenoptera. Recently, a new COI primer pair with a higher arthropod taxon recovery rate than the ZJB primers was developed, which had only a slight mismatch between the recovered read abundance and the mock community (ANML primers, Jusino et al., 2019). The ANML primers in avian insectivorous had a higher taxonomic coverage than the ZJB primers (Forsman et al., 2022). This highlights that primer choice and PCR conditions are crucial. The reported mismatches also indicate that to retrieve diet on the species level precise calibration is needed (see Krehenwinkel et al. (2017) for guidance).

For a validation study, we think that capturing the “real” arthropod prey community is an advantage. We detected a quantitative match between prey biomass and RRA on the taxonomic levels for which the camera data were reliable: order and (and to a lesser extent) family. We therefore conclude that our protocol allows for a quantitative use of RRA on the order and family level in insectivorous songbirds, and we think that especially the improved arthropod template‐primer match, the removal of uric acids from the DNA template, and the low “forgiving” annealing temperature in triplicate PCRs are important (see also Krehenwinkel et al., 2017). We recommend that future studies on insectivorous birds should test the modified Folmer‐Brown primers (this study) and the ANML primers (Jusino et al., 2019) and ideally include a validation with known diet to confirm this protocol indeed works with other types of insectivorous songbirds.

### 4.3 Approaches in sample collection and wild‐type diet assessment

#### 4.3.1 Sampling the full spectrum of wild‐type diets

In this validation study, we observed consumption of prey by the whole brood during the camera observations and collected feces for COI analysis of individual chicks, allowing for a variable temporal gap between observations and feces collection. An alternative calibration test is feeding individual birds a known diet after a period of food deprivation. The main disadvantage of such detailed calibrations in the case of arthropod diets is that they only include what prey are accessible to the researcher and what individuals want to eat in captivity. Therefore, we chose, and recommend, a calibration scheme that includes the full spectrum of the wild‐type diet, using random chicks in a brood and accepting that collected feces after camera sessions may also reflect what chicks have eaten earlier. Our study of 5–18 camera sessions each year showed that this a valid approach: COI metabarcoding of feces could quantitatively retrieve the observed diet, even when using aggregated fecal data. Moreover, in 2016, two fecal samples per nest were consistently collected right after the camera recording, but this did not improve the match between observed diet and COI data (Figure 7). This suggests that in this study, system diets are rather stable over days and within nests, which is supported by diets of multiple chicks across subsequent days within a nest being more similar than between nests (Nicolaus et al., 2019). The observed close quantitative match between prey communities in gizzard and intestine also supports the notion that diets at the level of individuals can be stable over some unit of time. Nevertheless, it remains to be tested what period of prey ingestion a single fecal sample represents.

#### 4.3.2 Reliability of taxonomic assignment and diversity in camera records

At the order level, diet composition was very similar between the methods, but at the family level, only within Lepidoptera, Diptera, and Coleoptera (Figure 7). Taxonomic assignments from camera footage was especially complicated for our observers in Hymenoptera, Hemiptera, and Araneae, leading to many “unknowns.” Quantitative comparisons between datasets of families within these orders could be improved when camera images would be analyzed by taxa experts. Nevertheless, small and inconspicuous species may remain difficult to identify from images. Also, providing adults often arrived with a beak filled with multiple prey items obscuring smaller species. Indeed, food items categorized as “unknown” on camera were usually described as small. An example of a common family in COI barcodes but not very often reported on camera were the dance flies (Empididae). However, also larger‐sized taxa were sometimes missed on camera, sensibly observers when unfamiliar with specific groups have classified those prey items at a higher taxonomic level only.

#### 4.3.3 Ecological limitations of metabarcoding

General limitations of using metabarcoding are that the life stage of prey cannot be assessed (larva vs. adult), and ingested items may not have been an intended food item. Indeed, parasites such as fleas, mites, and parasitoid wasps were common in COI barcodes, and especially Ichneumonidae parasitoid wasps (Figure 7). The RRA of Ichneumonid wasps was on average 0.94% but could be as high as 90% (Table S2). These wasps could have been ingested through their (caterpillar) hosts, but also directly because per camera session on average four (range 0–9) food items were assigned to Ichneumonidae. In the camera records, no other parasitoid wasp taxa were reported, and also no fleas or mites. In contrast, the COI reads contained one order of fleas (Siphonptera) and four orders of mites (Mesostigmata, Prostigmata, Sarcoptiformes, and Trombidiformes) (Figure 6). Siphonptera occurred with an average RRA of 0.01%, while mites, mostly Trombidiformes, had an average RRA of 0.4% (Figure 6). In summary, parasites in general contributed 1–2% to the diet, while being occasionally abundant in a single sample. We consider it unlikely that these parasites are more sensitive to PCR bias, and therefore think that they were ingested as secondary prey.

### 4.4 Taxonomic challenges in COI metabarcoding

#### 4.4.1 Public reference databases

Especially, in studies conducted in North America and Western Europe, sequencing the COI gene can obtain genus level identification of arthropods using public reference databases such as GenBank and the Barcoding of Life Database BOLD (King et al., 2015). We indeed used a public database for assignment of OTUs, but considered assignments to species level unreliable. Even at the family level, some taxa were assigned that do not occur in Europe (e.g., *Phanomorpha*) which again stresses that our validation set‐up is less precise at the family level. Only at the order level, we consider taxonomic assignment error of the retrieved COI reads unlikely. Nevertheless, two orders seen on camera as prey items were not detected in COI: Myriapoda (millipedes, centipedes, and others) and Orthoptera (grasshoppers, locusts, and crickets). In the case of the myriapods, the COI reads were assigned to another millipede order (Chordeumatida). As we did not taxonomically reassign prey items *a posteriori*, this was not corrected in our validation test. Orthoptera were very rarely provided prey items (Figure 6) and possibly therefore not detected. Orthoptera were detected in fecal samples of adult pied flycatchers using the same methodology (M. Tangili, pers. comm.) and were commonly observed as nestling prey in other flycatcher studies (Sanz et al., 2003). For more reliable assignment below order level, a reference barcode database of the local prey community should be established.

#### 4.4.2 Taxonomic overdominance in COI barcodes

Even though COI barcodes detected a large variety of taxa that matched with recorded diet, the taxonomic diversity of DNA barcodes in a study can potentially be biased (Alberdi et al., 2018; Krehenwinkel et al., 2017) especially when sample size is low. In our validation test, ca. 15% of the fecal samples were dominated by a single species. This overdominance could have been created during laboratory procedures but did not lead to a systematic overrepresentation of specific taxa. When subsampling feces for DNA‐extraction, a single large prey remain may lead to overrepresentation in DNA template. Additionally, PCR can cause overrepresentation of taxa, either randomly by template favoring after the initial PCR cycle, or non‐randomly, when primers may match better to some taxa than others. To avoid the risk of PCR bias, it is recommended to perform triplicate PCRs on each individual sample before pooling for sequencing (e.g., Vo & Jedlicka, 2014, but see Alberdi et al., 2018). We indeed executed PCR in duplicates or triplicates, and the repeated samples in a triplicate PCR set‐up in showed high repeatability of prey composition (Figure 3; Table 2). To avoid non‐random bias, using multiple markers is recommended because it increases the number of taxa recovered (Corse et al., 2019; da Silva et al., 2019). We decided against using multiple primer pairs because this jeopardized testing the relationship between RRA and the scaled biomass of dietary taxa. In our protocol, the primers did not systematically favor certain taxa and therefore non‐random bias is unlikely.

In conclusion, we propose that the observed overdominance in some samples may have been caused by transfer of larger fragments in feces to the DNA‐extraction. We reject the hypothesis that overdominance was an effect of prey digestion, for example, that degradation of DNA in the digestive track structurally varies between prey species, because we observed very similar prey communities in gizzard and intestine content. Dominance of single prey taxa could have been the result of birds consuming large amounts of a single prey species for some time, but that seems unlikely given the camera footage. Better insights into the potential causes of overdominance can come from comparing larger datasets, more detailed calibration feeding experiments, and laboratory study designs with more subsampled duplicates of feces.

### 4.5 Application to ecological studies

A clear added benefit exists of quantifying trophic interactions at the species level, rather than on higher taxonomic levels, as each species has its specific biology, including its niche, phenology, population dynamics, and adaptive capacities. Most traditional non‐molecular methods to quantify diets of generalist insectivorous organisms are unable to recognize lower taxonomic levels, and hence may miss important ecological interactions. Flycatchers do, for example, feed their nestlings with a rather large fraction of caterpillars (Burger et al., 2012), and the general caterpillar peak (measured by collecting caterpillar feces) has advanced in response to climate change (Both et al., 2009). However, little is known whether all caterpillar species do advance their phenology to a similar extent, and how flycatchers may switch from one caterpillar species to the other depending on its abundance and phenology. If we want to understand how food‐webs respond to climate or other environmental changes including its eco‐evolutionary dynamics, we need to quantify interactions preferably on the species level, because population and evolutionary dynamics are species characteristics. The accuracy of metabarcoding techniques to identify trophic interactions at the species level may not yet be absolute, but often much better than traditional methods. Furthermore, it allows to study interactions beyond the easily observed nestling periods, and, for example, we may now quantify year‐round diet as long as we can obtain feces of the species of interest.

Metabarcoding may hence be a quantitative tool to study the relative contribution of different prey taxa in the diet of consumers, but as individuals do not survive on relative contributions but rather need absolute amounts of food, it is still necessary to measure intake rates to study how diet composition changes depending on ecological circumstances. Hence, we see this method as an important addition to the toolkit of field ecologists, enabling them also to focus more on sampling the temporal and spatial abundance of the prey that really are important for the predator species of interest.

## AUTHOR CONTRIBUTIONS

**Yvonne I. Verkuil:** Conceptualization (equal); Data curation (equal); Formal analysis (lead); Funding acquisition (equal); Methodology (lead); Project administration (equal); Visualization (lead); Writing – original draft (lead). **Marion Nicolaus:** Conceptualization (supporting); Data curation (equal); Methodology (equal); Supervision (equal); Writing – review & editing (equal). **Richard Ubels:** Data curation (lead); Investigation (equal); Methodology (equal); Project administration (equal); Writing – review & editing (equal). **Maurine W. Dietz:** Conceptualization (equal); Formal analysis (equal); Funding acquisition (equal); Methodology (equal); Supervision (equal); Writing – review & editing (equal). **Jelmer M. Samplonius:** Formal analysis (equal); Investigation (equal); Writing – review & editing (equal). **Annabet Galema:** Investigation (equal); Methodology (supporting); Writing – review & editing (equal). **Kim Kiekebos:** Formal analysis (supporting); Methodology (supporting); Software (equal); Writing – review & editing (equal). **Peter de Knijff:** Conceptualization (equal); Methodology (equal); Resources (lead); Supervision (equal); Writing – original draft (equal). **Christiaan Both:** Conceptualization (lead); Formal analysis (supporting); Funding acquisition (lead); Investigation (equal); Methodology (equal); Supervision (equal); Writing – original draft (equal).

## Supporting information

Appendix S1

## Data Availability

Raw data (Fastq files) are archived at University of Groningen. Data table and scripts are deposited with Dryad at Verkuil (2021). This collection contains (a) Data tables: OTUtable, TAXAtable, SAMPLE‐INFO, and (b) scripts: USearch jobscript, Rgui‐scripts.
