# Supplementary material for: DNA metabarcoding quantifies the relative biomass of arthropod taxa in songbird diets: Validation with camera‐recorded diets
Source: Ecol Evol. 2022 May 6;12(5):e8881. doi: 10.1002/ece3.8881 (PMC9077022; doi:10.1002/ece3.8881)
Supplement: Supplementary file 1 — Appendix S1 [file ECE3-12-e8881-s001.docx]

**APPENDIX**

**TABLE** **S1** Overview of the 20 OTUs detected in the pooled negative PCR controls (NC, sequence ID T0820). For each detected OTU, we show GenBank accession number of the closest match, the number of reads, and the taxonomic assignment.

| **ID = T0820** | **Accession nr. Match GenBank** | **N reads in NC** | **Organism** | **Kingdom** | **Phylum** | **Class** | **Order** | **Family** | **Genus** |
| --- | --- | --- | --- | --- | --- | --- | --- | --- | --- |
| OTU NC_18 | KM449858 | 1 | *Lochmaea capreae* | Animalia | Arthropoda | Insecta | Coleoptera | Chrysomelidae | Lochmaea |
| OTU NC_3 | KU917964 | 7 | *Strophosoma capitatum* | Animalia | Arthropoda | Insecta | Coleoptera | Curculionidae | Strophosoma |
| OTU NC_6 | KX844104 | 5 | *Campylocheta praecox* | Animalia | Arthropoda | Insecta | Diptera | Tachinidae | Campylocheta |
| OTU NC_20 | KM023121 | 1 | *Kleidocerys resedae* | Animalia | Arthropoda | Insecta | Hemiptera | Lygaeidae | Kleidocerys |
| OTU NC_5 | KR897618 | 6 | *Diploplectron sp.* | Animalia | Arthropoda | Insecta | Hymenoptera | Crabronidae | Diploplectron |
| OTU NC_2 | KX665108 | 9 | *Formica sanguinea* | Animalia | Arthropoda | Insecta | Hymenoptera | Formicidae | Formica |
| OTU NC_9 | KR878219 | 3 | *Glypta sp.* | Animalia | Arthropoda | Insecta | Hymenoptera | Ichneumonidae | Glypta |
| OTU NC_12 | MG349058 | 2 | *Pleolophus sp.* | Animalia | Arthropoda | Insecta | Hymenoptera | Ichneumonidae | Pleolophus |
| OTU NC_19 | KX043145 | 1 | *Noctua fimbriata* | Animalia | Arthropoda | Insecta | Lepidoptera | Noctuidae | Noctua |
| OTU NC_17 | LK077046 | 1 | *Apteryx australis* | Animalia | Chordata | Aves | Apterygiformes | Apterygidae | Apteryx |
| OTU NC_15 | GU571396 | 1 | *Ficedula hypoleuca* | Animalia | Chordata | Aves | Passeriformes | Muscicapidae | Ficedula |
| OTU NC_10 | KP719757 | 2 | *Sphyrna lewini* | Animalia | Chordata | Elasmobranchii | Carcharhiniformes | Carcharhinidae | Sphyrna |
| OTU NC_14 | AY049855 | 1 | *Scyliorhinus torazame* | Animalia | Chordata | Elasmobranchii | Carcharhiniformes | Scyliorhinidae | Scyliorhinus |
| OTU NC_7 | AF212180 | 5 | *Triakis semifasciata* | Animalia | Chordata | Elasmobranchii | Carcharhiniformes | Triakidae | Triakis |
| OTU NC_8 | KF808207 | 5 | *Gymnura altavela* | Animalia | Chordata | Elasmobranchii | Myliobatiformes | Gymnuridae | Gymnura |
| OTU NC_11 | KU705517 | 2 | *Rhinoptera bonasus* | Animalia | Chordata | Elasmobranchii | Myliobatiformes | Myliobatidae | Rhinoptera |
| OTU NC_16 | KY176594 | 1 | *Rhinoptera marginata* | Animalia | Chordata | Elasmobranchii | Myliobatiformes | Myliobatidae | Rhinoptera |
| OTU NC_4 | KY176593 | 6 | *Glaucostegus cemiculus* | Animalia | Chordata | Elasmobranchii | Pristiformes/Rhiniformes | Rhinobatidae | Glaucostegus |
| OTU NC_13 | KY176593 | 1 | *Glaucostegus cemiculus* | Animalia | Chordata | Elasmobranchii | Pristiformes/Rhiniformes | Rhinobatidae | Glaucostegus |
| OTU NC_1 | NG_046424 | 10 | *Homo sapiens* | Animalia | Chordata | Mammalia | Primates | Hominidae | Homo |
| **Total** |  | **70** |  |  |  |  |  |  |  |
